# High Rates of Bacterial Pulmonary Co-Infections and Superinfections Identified by Multiplex PCR among Critically Ill COVID-19 Patients

**DOI:** 10.3390/microorganisms9122483

**Published:** 2021-11-30

**Authors:** Regev Cohen, Frida Babushkin, Talya Finn, Keren Geller, Hanna Alexander, Candice Datnow, Martina Uda, Maurice Shapiro, Svetlana Paikin, Jonathan Lellouche

**Affiliations:** 1 Adelson School of Medicine, Ariel University, Ariel 4077625, Israel; tfinnfried@laniado.org.il (T.F.); jlellouche@laniado.org.il (J.L.); 2 Infectious Diseases Unit, Sanz Medical Center, Laniado Hospital, Netanya 4244915, Israel; fbabushkin@laniado.org.il (F.B.); keren.geller@gmail.com (K.G.); hrezmovich@laniado.org.il (H.A.); cdatnow@laniado.org.il (C.D.); 3 Intensive Care Unit, Sanz Medical Center, Laniado Hospital, Netanya 4244915, Israel; muda@laniado.org.il (M.U.); mshapiro@laniado.org.il (M.S.); 4 Clinical Microbiology Laboratory, Sanz Medical Center, Laniado Hospital, Netanya 4244915, Israel; svetlana@laniado.org.il

**Keywords:** COVID-19, co-infection, superinfection, community-acquired pneumonia, ventilator-associated pneumonia, FimArray, PCR, critically ill, intensive-care unit

## Abstract

Background: The role of bacterial co-infection and superinfection among critically ill COVID-19 patients remains unclear. The aim of this study was to assess the rates and characteristics of pulmonary infections, and associated outcomes of ventilated patients in our facility. Methods: This was a retrospective study of ventilated COVID-19 patients between March 2020 and March 2021 that underwent BioFire^®^, FilmArray^®^ Pneumonia Panel, testing. Community-acquired pneumonia (CAP) was defined when identified during the first 72 h of hospitalization, and ventilator-associated pneumonia (VAP) when later. Results: 148 FilmArray tests were obtained from 93 patients. With FilmArray, 17% of patients had CAP (16/93) and 68% had VAP (64/93). Patients with VAP were older than those with CAP or those with no infection (68.5 vs. 57–59 years), had longer length of stay and higher mortality (51% vs. 10%). The most commonly identified FilmArray target organisms were *H. influenzae, S. pneumoniae, M. catarrhalis* and *E. cloacae* for CAP and *P. aeruginosa* and *S. aureus* for VAP. FilmArray tests had high negative predictive values (99.6%) and lower positive predictive values (~60%). Conclusions: We found high rates of both CAP and VAP among the critically ill, caused by the typical and expected organisms for both conditions. VAP diagnosis was associated with poor patient outcomes.

## 1. Introduction

Patients hospitalized in intensive care units (ICUs) with critical novel coronavirus disease 2019 (COVID-19) have a high mortality rate, reaching ~50% (range 16–78%) [1,2]. The causes contributing to mortality are respiratory failure with severe hypoxemia and its immediate consequences, multiorgan failure, thromboembolism, hemorrhage, and healthcare-associated infections (HAIs) associated with bacteria and filamentous fungi. Bacterial co-infections, diagnosed around the time of severe acute respiratory syndrome coronavirus 2 (SARS-CoV-2) infection, appear to be uncommon, occurring in 0.6 to 3.2% of patients [3], although some studies reported higher rates, reaching 26–28% [4,5,6] or more [7]. The co-infection rates in COVID-19 seem to be lower than those reported for influenza pandemics [8,9]. The main pathogens associated with co-infections are *S. aureus*, *S. pneumoniae* and *H. influenzae* [4,6,10], while in some studies *S. pneumoniae* takes a minor role [7,8,10,11,12].

As the pandemic unfolds and more data come to light, the role of secondary infections, i.e., HAIs, among critical COVID-19 patients in the ICU, becomes more pronounced. Superinfection rates in ICUs were reported in 13.5–48% of patients [10,13,14,15], and most reported cases of HAIs include ventilator-associated pneumonia (VAP), with *P. aeruginosa* the main causative agent [10]. Superinfection may be becoming more of an issue now because of increased length of ICU stay secondary to improved overall COVID-19 survival rates, the now universal use of corticosteroid therapy and increased use of other immunomodulatory drugs such as tocilizumab and baricitinib, and improved reports from focused studies conducted to assess COVID-19-related infectious complications. Outbreaks of multidrug resistant bacteria are also commonly reported, including a variety of organisms such as carbapenem resistant *Acinetobacter baumannii*, carbapenem resistant Enterobacterales, multidrug resistant *Pseudomonas aeruginosa*, methicillin resistant *Staphylococcus aureus* (MRSA), *Candida auris* and others [2,16,17,18]. Since most COVID-19 patients in the ICUs are treated empirically with antimicrobials [4,19], antibiotic stewardship programs along with enhanced infection control protocols are essential in controlling the emergence of these dreaded infections. 

Israel is currently (October 2021) in the midst of the fourth COVID-19 wave. The three previous waves occurred between March 2020 and March 2021. Very few clinical data have emerged from Israel during this period of time regarding critically ill COVID-19 patients, rates of co-infection and superinfection, or their outcomes. In this study, we primarily aimed to analyze these aspects retrospectively among COVID-19 patients who were admitted to Sanz Medical center and needed mechanical ventilation. We focused on data from a rapid multiplex polymerase chain reaction (mPCR) kit for the molecular identification of lower respiratory tract pathogens (Biofire^®^, FilmArray^®^ Pneumonia Panel, bioMérieux, Marcy-l’Étoile, France), as well as, and in comparison with, classical microbiology methods. Our secondary aims were to assess the antimicrobial decisions related to the results of this rapid test in the context of an antimicrobial stewardship program. 

## 2. Materials and Methods

### 2.1. Study Design

This was a retrospective observational study including patients hospitalized between March 2020 and March 2021 in the COVID-19 ICU of Sanz Medical Center, a university-affiliated, 400-bed regional hospital, located in the city of Netanya, Israel.

### 2.2. Patient Selection

COVID-19 patients who were mechanically ventilated at some point during their hospitalization course were included in the study if they underwent FilmArray^®^ (FA) testing. FA tests were conducted at physicians’ discretion with infectious diseases expert approval. Tests were used to assist physicians in the diagnosis of a secondary community-acquired pneumonia (CAP, when suspected during the first 72 h from admission), or VAP (after 72 h from admission). PCR and cultures were obtained when patients showed signs of clinical and respiratory deterioration; when they developed sepsis or septic shock; when bacterial infection was suspected (recurring fever, the appearance of a new infiltrate on imaging or elevation of inflammatory indexes) and typically, immediately after intubation that facilitated the acquisition of respiratory samples.

### 2.3. Microbiology Samples

FA tests were performed on sputum samples, endotracheal aspirates (ETA, suctioned from intubated patients), non-bronchoscopic lavage (NBL) fluid and from bronchoalveolar lavage (BAL, when performed by a pulmonologist) in accordance with the manufacturer’s instructions. The results were directly reported to the treating physician and to the infectious diseases expert that requested the test. The same specimens that were used for FA testing were typically also used for conventional microbiology methods. In cases for which this was unavailable, new samples were requested. For comparisons between the FA results and microbiology cultures, we included only cases for which the same sample was used for both analyses or the time interval between FA and culture samples was ±2 days.

Samples were performed, analyzed and results reported in accordance with the Clinical Microbiology Procedures Handbook [20]. Samples were inoculated on blood (trypticase soy agar enriched with 5% sheep blood), chocolate blood, Columbia nalidixic acid, MacConkey, CHROMagar^™^ Orientation agars (Hy Laboratories, Rehovot, Israel). Suspected colonies were identified using a VITEK^®^ 2 system (bioMérieux, Marcy-l’Étoile, France). Antibiotic susceptibility testing was performed using a VITEK^®^ 2 system and confirmed by disk diffusion. Susceptibility was interpreted according to current CLSI guidelines [21]. Methicillin (oxacillin) resistance in *S. aureus* was determined by resistance to cefoxitin by disk diffusion and confirmed by the presence of the SCC*mec/mecA* genetic elements [21].

COVID-19 was detected using PCR platforms (Xpert^®^ Xpress SARS-CoV-2, Cepheid, Sunnyvale, CA, USA; BD SARS-CoV-2, Beckton Dickinson, Franklin Lakes, NJ, USA; Allplex™ 2019-nCoV Assay, Seegene Inc, Seoul, Korea).

### 2.4. Evaluation of FA Results as Positive

Since FA test results are reported with a quantitative assessment of the DNA load, the source of the sample (sputum vs. ETA, NBL or BAL) is important to assess the relevance of the positive result. For this retrospective analysis we used two sets of rules to assign a positive FA result as such. A strict set was used with thresholds of: ≥10^4^ genome copies/mL for BAL samples, ≥10^5^ for ETA and NBL samples and 10^7^ for sputum samples as positive. A second less strict set was used: ≥10^4^ for BAL, ETA and NBL and ≥10^6^ for sputum samples.

For comparison between cultures and FA tests we considered all the microorganisms identified in each culture, with the exclusion of *Candida* species and *Enterococcii*, which were regarded as contamination. Sensitivity and specificity were calculated using culture as gold standard.

### 2.5. Statistical Analysis

Categorial variables were expressed as frequencies and percentages and compared using Fishers’ exact test. Continuous variables were presented as means and standard deviations (SD) or medians and interquartile range (IQR) and compared using Student’s *t*-test. Calculations were performed using GraphPad Prism^®^ v.7.0(GraphPad Software LLC, San Diego, CA, USA). Statistical significance was defined when *p* < 0.05.

The study was authorized by the Institutional Review Board (IRB) of Sanz medical center, 0014-21-LND.

## 3. Results

### 3.1. Patients

Between March 2020 and March 2021, 887 COVID-19 patients were hospitalized in our facility, of which 132 (15%) needed mechanical ventilation at some point during their hospital stay and, of these, 93 (70%) were tested for secondary bacterial infection using the FA pneumonia panel. Of these 93 patients, 49 had one test performed during their hospitalization, 35 had two tests, seven had three tests and two had four tests; altogether 148 FA tests were included in the analysis. In most cases, each FA represented a situation in which the patient was suspected of having a bacterial infection. There were few specific cases in which young critically ill patients (for example, peri-partum ventilated women) underwent more frequent testing, especially when they were not treated with empiric antimicrobials. The mean (SD) and median (IQR) interval between repeated FA tests were 9.9 (8.2) and 8 (5–12) days, respectively.

The mean (SD) and median (IQR) patients’ age were 64.6 (15.1) and 67 (56–76.5) with male predominance (70%). Long term care facility residency was rare (5/93, 5.3%). The mean (SD) duration from COVID-19 diagnosis to hospitalization was 3.8 days (3.7) with median (IQR) of 3 days (0–6), while six patients were diagnosed with COVID-19 during their hospitalization. Mean and median length of stay (LOS) were 28.9 (17.3) and 24 days (17–38).The mean was 32.9 days for males and 19.7 days for females, *p =* 0.0006. In-hospital mortality was 36/93 (38.7%) and mean (SD) and median (IQR) interval from admission to death were 30.6 days (19.9) and 25 (16–39.7).

### 3.2. CAP and VAP Diagnosis

Forty patients were tested during the first 72 h of hospital stay for CAP diagnosis. The mean (SD) and median (IQR) interval from admission to first FA test were 1.3 (0.6) and 1 (1–2) days. Of the 93 patients, 16 (17%) had CAP, when considering the less strict set of rules. Most of these patients (15/16, 94%) were treated with antimicrobials directed for CAP and in 6/16 (37.5%) there was a concomitant positive culture result. With the strict set of rules, there were 12/93 (13%) positive FA results, of which six (50%) also had a positive culture result.

74 patients were tested for VAP (including 21 patients also tested for CAP and 53 that were tested only for VAP). The mean (SD) and median (IQR) interval from admission to first FA directed for VAP diagnosis were 8 (6.7) and 6 (4–9) days. With the less strict rules, 64/93 patients (68%) had VAP (58 patients with VAP only and 6 also had CAP).

The 58 patients tested for VAP had altogether 108 FA tests. 75/108 (69%) were FA-positive, and 33 tests were FA-negative. In 62/75 cases (82%) there was also a concurrent positive culture and in 51/75 cases (68%) the results triggered antibiotic therapy change (commencing treatment or either upgrading or downgrading a given drug) to treat VAP.

With the strict set of rules, there were 62/108 (57%) positive FA tests and in 53/62 (85%) a positive culture was recorded. More data are shown in Table 1 and Table 2.

### 3.3. FA Results

Most FA tests were performed on ETA samples (102/148, 69%), and the rest were: 22 sputa (15%), 16 BAL (11%) and 8 NBL (5%).

For the strict set of rules for evaluation of FA results, positive rates were higher for tests performed for VAP vs. CAP diagnosis (62/108 (57%) vs. 12/40 (30%), (odds ratio (OR) 0.31, 95% confidence interval (CI) 0.14–0.7, *p =* 0.005). The most common bacterial targets were *P. aeruginosa* (27 samples, 36.4%), *S. aureus* (24 samples, 32.4%)—12 methicillin-susceptible *S. aureus* (MSSA) and 12 methicillin-resistant *S. aureus* (MRSA), and *H. influenzae* (15 samples, 20.2%).

Twelve FA tests were considered positive for CAP diagnosis. Of these 12, the most common targets were *H. influenzae* (7/12, 58.3%), *S. pneumoniae* (6/12, 50%) and *M. catarrhalis* and *E. cloacae* (2/12, 16%, each). Of the 62 positive FA tests performed for VAP, the most common targets found were: *P. aeruginosa* (26/62, 42%), *S. aureus* (23/62, 37%, 12 MRSA and 11 MSSA), *K. pneumoniae* (14/62, 22.5%), and *H. influenzae* (8/62, 13%), Table 3.

**Figure 1:**
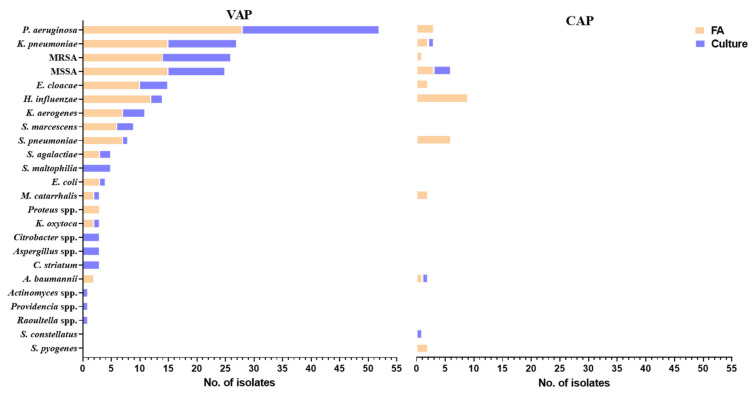
Absolute number of isolates identified by FA and standard cultures, in patients with CAP and VAP.

For the less stringent set of rules, positive results were more common: 91/148 (61%); 16/40 (40%) for CAP and 75/108 (69%), OR = 0.29, 95% CI = 0.13–0.64, *p =* 0.002. The same patterns of bacterial distribution as in the strict set were found and are depicted in Table 3 and Figure 1.

Three FA tests were positive for viruses: two were positive to *Rhinovirus/Enterovirus* and one for *Parainfluenza* virus. CTX-M was positive in 17 and 20 FA results (including one case of sputum 10^4^ and one of NBL 10^5^) according respectively to the two set of rules. None of the resistance genes were detected among the tests performed for CAP diagnosis.

### 3.4. Associated Outcomes Related to FA Results

Patients with VAP diagnosis were significantly older than those with CAP or those with neither diagnosis (median age 68.5 vs. 57 and 59, *p =* 0.025 and *p =* 0.001, respectively). These patients also had longer hospitalization duration medians (29 vs. 22.5 and 17 days, *p =* 0.08 and *p =* 0.003). In-hospital mortality was 1/10 and 2/19 (10%) among patient with CAP or with neither diagnosis respectively, vs. 33/64 (51.5%) in patients with VAP (*p =* 0.001) (Table 2).

### 3.5. Culture Results

Out of 148 FA tests, for 128 (86%) there was a comparable microbiologic culture, of which a total of 105 bacteria/fungi were identified. In 54 cases the cultures were negative, one microorganism was cultured in 44 cases, two microorganisms in 30 cases and three bacteria in one case. ETA was the source of the culture in 88/128 cases (68%), and the rest were: 16 BAL, 16 sputa and eight NBL. The bacteria found were: 24 *P. aeruginosa*, 13 MSSA, 13 *K. pneumoniae*, 12 MRSA, 8 *E. cloacae*, five *S. maltophilia*, four *K. aerogenes*, three *Citrobacter* spp., three *Aspergillus* spp. and 20 other bacteria (Figure 1).

### 3.6. Comparison between FA and Culture Results

Of the 128 comparable FA-culture couples, 46 were FA-negative of which 38 (82%) were also culture-negative. Of the 82 FA-positive samples, 66 (80%) were also culture positive (the same pathogens appearing as in the FA panel results in 60/82, 73%). Of the 16 FA-positive/culture negative cases, eight were treated with antimicrobials at sampling time, a fact that could affect culture positivity. Interestingly, in four of these 16 cases, a subsequent culture was positive for the same microorganism that was previously detected in the FA panel (1 each: *E. cloacae, K. pneumoniae, H. influenzae and S. pyogenes*).

We evaluated the performance of FA as compared with the gold standard of classical microbiology cultures using the strict and less strict rules (Table 4). By applying the strict rules, the overall sensitivity and specificity of the FA test were 78.4% and 98.1% respectively, with positive and negative predictive values (PPV and NPV) of 66.3% and 98.9%. With less strict panel of rules, the sensitivity increased to 92.2% with a reciprocal reduction in the specificity to 96.6%. The lowest sensitivities were notable in cases of *H. influenzae, A. baumannii, E. cloacae* and MSSA

In 17 samples, the cultures were positive for bacteria that were not part of the FA panel, and these included five *S. maltophilia,* three *Aspergillus* spp., three *C. striatum*, two *Citrobacter* spp. and four others. One *Providencia stuartii* was cultured while the FA detected *Proteus* spp., probably a misidentification of the FA panel.

The sensitivity, specificity, PPV and NPV for CTX-M detection as compared with phenotypic culture results, using the less strict rules, were 82.3%, 94.6%, 70% and 97.2%, respectively (Table 4). One case of NDM was detected by the FA panel with no phenotypic counterpart in the respiratory or gastrointestinal tracts. No other resistant genes were detected.

### 3.7. Effect of FA Results on Antimicrobial Therapy

In 107/148 (72%) cases, FA results influenced antimicrobial decisions including avoidance of using antimicrobials, starting, stopping, upgrading or downgrading an already given treatment. In 41 cases (27%) it triggered the initiation of treatment—34 cases that were not previously treated and seven cases that were previously treated but were at the time on “antimicrobial treatment window”. 18/34 cases were treated for community-acquired organisms with ceftriaxone, four were given MSSA-directed therapy. All seven patients that were re-treated were given extended spectrum antimicrobials, that included coverage for MRSA, Extended-spectrum beta-lactamase (ESBL) producing Enterobacterales and *P. aeruginosa*. Antimicrobials were stopped in 13 cases, 11 of which had negative FA tests, and in two cases with positive FA results. Antimicrobials were avoided in 21 cases, all of which had negative FA results (Table 5).

Downgrading the coverage spectrum was noted in five cases, and upgrading in 27 cases, mostly for *P. aeruginosa* coverage (*n* = 13), as well as for MRSA and ESBLs (four each). In 41 cases, FA results did not affect the given treatment, 17/41 had negative FA, but antimicrobials were still considered appropriate, and in the rest the patients were already covered with the presumed antimicrobials for the identified FA targets found (Table 6).

Of the 57 negative or insignificant FA results, in 34 (59%) these results were associated with stopping, downgrading or withholding antimicrobial therapy. In six cases, negative FA results triggered commencing antimicrobial therapy (two ceftriaxone and one cefazolin) or upgrading it (one from cefazolin to trimethoprim/sulfamethoxazole, one from azithromycin to ciprofloxacin and one from colistin to vancomycin and amphotericin B).

### 3.8. Effect of Post-FA Culture Result on Antimicrobial Therapy

Most cases of negative FA tests were confirmed by negative culture results (38/46, 82%). Among the eight discordances, there were four cases that were not covered until the result of the culture (one ceftriaxone-susceptible *E. cloacae*, one *H. influenzae*, one *S. maltophilia* and 1 *Aspergillus flavus*). The other four cases (one *C. striatum*, one MSSA, 1 *Actinomyces* spp. and 1 *S. constellatus*) had already been treated empirically. Two cases of ceftriaxone-susceptible *Citrobacter* spp. (an organism that does not appear in the FA panel), were seen in cultures, but both cases were adequately treated empirically.

## 4. Discussion

The main findings of this study are the relative high rates of both co-infections and superinfections among critically ill, ventilated COVID-19 patients and the association between the occurrence of positive FA results and poor patients’ outcomes. In concurrence with recent literature [4,5,6], we also encountered high rates of positive mPCR tests from COVID-19 patients’ secretions (mostly ETAs). Depending on the strictness of bacterial DNA loads evaluation, we had 13–17% positive FA results for patients with CAP and 68% positive FA results for VAP. It is important to note that a considerable part (50–65%) of these positive early FA test results for CAP were not met with positive cultures later, suggesting, at least in part, low bacterial loads probably representing bacterial colonization or non-viable bacteria.

High rates of COVID-19-bacterial co-infections identified with molecular methods among COVID-19 patients were reported recently by our group [7] and others [5,12,22,23]. The bacteria encountered by the tests performed on admission were typical for community-acquired infections, including mainly *H. influenzae, S. pneumoniae and M. catarrhalis*. VAP was significantly more prevalent than CAP, occurring in 62% of the cohort, and with better correlation to the microbiology results. As was previously reported [10,24], *P. aeruginosa* and *S. aureus* including MRSA, MSSA and Enterobacterales were the most common microorganisms seen in this study. A recent Italian study prospectively followed 248 patients in eight ICUs, and found 36% secondary bacterial infection, primarily VAP, with the same dominant bacteria as were reported in our study but without the use of molecular diagnostic methods [25].

Mortality among critically ill, ventilated COVID-19 patients is high. In our cohort, 38% of patients died, which is lower than typically reported, although the range varies considerably [26]. In our study, mortality was considerably lower (10%) among ventilated patients who were diagnosed with CAP (without subsequently also developing VAP) and among those who had only negative FA results. This was in clear contrast to the 51% mortality rate among patients with VAP diagnosis based on positive FA tests. Thus, VAP may be a surrogate for mortality, although causality should not be readily assumed. The patients with VAP in our cohort were older by nearly a decade as compared to those with CAP or neither diagnosis, and older age, typically associated with higher rates of comorbidities, is a very strong independent predictor of death from COVID-19 [26,27,28]. Hence, older, more fragile and pre-morbid patients, had longer duration of ICU stay and consequently more exposure to other infectious and non-infectious ICU-related complications. Aside from the severe pulmonary failure associated with SARS-CoV-2 infection, these patients suffered a myriad of complications due to prolonged ICU stay, including bleeding diathesis, arterial and venous thromboembolic events, spontaneous and iatrogenic pneumothorax and pneumomediastinum, and other HAIs including central line related bloodstream infections. Typically, patients succumbed to these complications (and primarily to infectious causes) and not to the acute respiratory distress syndrome (ARDS) associated with COVID-19. A prospective study on 45 critically ill patients in Switzerland in which 478 lower respiratory samples were assessed longitudinally, has reported 42% respiratory superinfections and association with poor outcomes, including longer ICU stay and lower ventilator-free survival rate. In this cohort, there were no differences between the patients with superinfection and those without, including their age and comorbidities, implying that the superinfection itself may be the surrogate for unfavorable consequences [29].

HAIs among COVID-19 patients are commonly caused by multidrug resistant organisms (MDROs) and filamentous fungi. In our patients, *P. aeruginosa, S. aureus* (MRSA, but also MSSA) and Enterobacterales were the main causes of VAP, identified by FA tests, but *S. maltophilia* and *Aspergillus* spp. were also encountered, posing a great treatment challenge. The short turn-around time of diagnosing a MDRO, using FA as opposed to classical methods, confers significant advantages in both providing an immediate, tailored antibiotic therapy to cover an unexpected organism (as MRSA or *A. baumannii*), avoiding the empiric use of wide-spectrum combination therapy for a deteriorating critically ill patients while awaiting Gram-stains results (which could be more difficult to obtain in laboratories without the means to inactivate infectious materials), and implementing contact precautions when a suspected organism or resistance mechanism appear in the FA panel result. On the other hand, care should be applied not to automatically withdraw antibiotic therapy in response to negative FA results, since important organisms may be missing from the panel (such as *Citrobacter* spp., *S. maltophilia* and others, including fungi). Discrepancies between the FA panel and cultures should also be considered.

The performance of the FA kit in our study was comparable to previous reports [30]. Using the less strict set of rules, we found that the overall NPV was 99.6% and was similar between Gram-negative and Gram-positive bacteria. Hence, a negative FA result in a critically ill patient could be relied on, and unnecessary antibiotic therapy withdrawn, as long as pathogens that do not appear in the FA panel are taken into consideration. The PPV using these rules was 57.6%, representing alleged relatively high rates of false-positive FA results, that could result in antimicrobial overuse. It should be noted that specifically for *P. aeruginosa*, which was the most common organism in our cohort, the PPV was the highest (92.3%). When stricter rules were applied, the sensitivity decreased and the PPV increased to 66.3%, with slight decrease in the NPV (to 99%). Specifically for *P. aeruginosa* the PPV in this setting was 100%. In real-life, many infectious diseases experts would probably choose not to ignore a positive ETA sample for MRSA, for example, because only 10^5^ copies/mL and not 10^6^ copies/mL were reported.

This study has limitations. The cohort of patients was small, and it was conducted in a single, medium-sized single medical center. Nevertheless, this cohort represents most of the ventilated COVID-19 patients seen in this facility during three pandemic phases. In this study, FA tests and cultures were not always performed on the same samples, which could have an impact on the comparative performance results. However, out of 128 comparative samples, in 85 (66%) the samples used were identical, and in 30 (23%) the culture was taken before FA testing—hence antimicrobials, if administered in the interval, should not have influenced the culture results, and may have had only minor effects on FA tests. In 13 cases (10%) the cultures were taken a day or two after FA testing and in all but three cases there was still a perfect correlation between the tests. In two of the three uncorrelated cases, the cultures grew MSSA and *H. influenzae* while the FA was negative, and in only one case *K. pneumoniae* detected by FA did not grow in culture. We did not collect data on the co-morbidities of the patients and other related data concerning other infectious and non-infectious complications during the ICU stay. Similarly, we did not conduct a multi-variate analysis for the mortality causes.

To conclude, we have shown high rates of both co-infection (CAP) and superinfection (VAP) among critically ill, ventilated COVID-19 patients caused by the typical and expected organisms for both conditions. The patients suffering from VAP were older and had longer ICU stays and mortality rates, as compared to those having only CAP or not having any positive FA test results. The correlation of FA tests with classical cultures was higher for VAP patients, with very high NPV and acceptable PPV, especially for *P. aeruginosa*. The timely accessible results of FA tests had an impact on antibiotic stewardship decisions, with negative results typically assisting in termination of therapy while positive results assisted in expanding or commencing antibiotic therapy. The performance of the FA tests was comparable to previous studies.

## Figures and Tables

**Table 1:** Ventilated COVID-19 patients and microbiology samples characteristics.

Patients’ Characteristics	Number, Median (IQR) Total
Patients (*n* = 93)	
Age, years	67 (56–76.5)
Male (%)	65 (70)
LTCF residence, number (%)	5 (5.3)
Length of stay, days	24 (17–38)
COVID-19 diagnosis to hospitalization *, days (*n* = 87)	3 (0–6)
Hospitalization to FA testing, days	3 (1.5–6)
For diagnosis of CAP, days (*n* = 40)	1 (1–2)
For diagnosis of VAP, days (*n* = 53)	6 (4–9)
Patients diagnosed with ** (%):	
CAP	16 (17)
VAP (without CAP)	58 (62)
Neither	19 (20)
C-reactive protein, mg/dL	133 (76–220)
Mortality (%)	36 (38.7)
Hospitalization to death, days (*n* = 36)	25 (16–39.7)
VAP *** diagnosis to death, days (*n* = 33)	17 (8.5–35.5)
Molecular and microbiology samples	
FA samples *n* = 148 (%)	
ETA	102 (69)
Sputum	22 (15)
BAL	16 (11)
NBL	8 (5)
Cultures samples *n* = 128 (%)	
ETA	88 (69)
Sputum	16 (12.5)
BAL	16 (12.5)
NBL	8 (6)

* Six patients were diagnosed with COVID-19 while hospitalized; ** using the less strict set of rules; *** including 6 patients who were also previously diagnosed with CAP. Abbreviations: IQR—interquartile range; LTCF—long term care facility; FA—FilmArray; CAP—community acquired pneumonia; VAP—ventilator associated pneumonia; ETA—endotracheal aspirate, BAL—bronchoalveolar lavage; NBL—non-bronchoscopic lavage.

**Table 2:** Comparisons between patients’ characteristics and outcomes according to diagnosis of pneumonia using the less strict set of rules.

	Patients with Only CAP *n* = 10	Patients with VAP * n = 64	Patients with Neither CAP nor VAP, *n* = 19	CAP vs. VAP * p (95% CI ** or OR ***)	CAP vs. Neither *p* (95% CI or OR)	VAP * vs. Neither *p* (95% CI or OR)
Age (years); median (IQR)	57 (48.2–67.5)	68.5 (63–77)	59 (39–69)	0.025 (1.1 to 17.5)	NS	0.001 (−19.6 to −5)
Male (%)	6 (60)	48 (75)	12 (63)	NS	NS	NS
LTCF residence (%)	0	5 (7.8)	0	NS	NS	NS
Length of stay-days, median (IRQ)	22.5 (16.2–29.5)	29 (21–42.2)	17 (12–29)	0.08 (−1.3 to 23.3)	NS	0.003 (−23.3 to −4.7)
Mortality ** (%)	1 (10)	33 (51.5)	2 (10.5)	0.001 (9.5)	NS	0.0014 (9)

* Including 6 patients who also had CAP. ** using student’s *t*-test. *** using Fisher’s exact test. Abbreviations: CAP—community acquired pneumonia; VAP—ventilator-associated pneumonia; OR—odds ratio; CI—confidence interval; IQR—interquartile range; LTCF—long term care facility; NS—nonsignificant.

**Table 3:** Bacteria identified by FA tests using two sets of rules among patients with CAP and VAP.

	Strict Rules *			Less Strict Rules **		
	CAP (*n* = 12)	VAP (*n* = 62)	Total (*n* = 74)	CAP (*n* = 16)	VAP (*n* = 75)	Total (*n* = 91)
Gram negative, number of targets detected (% ***)						
*E. cloacae*	2 (16.6)	6 (9.6)	8 (10.8)	2 (12.5)	10 (13.3)	12 (13.1)
*E. coli*	0	2 (3.2)	2 (2.7)	0	3 (4)	3 (3.2)
*A. baumannii*	1 (8.3)	1 (1.6)	2 (2.7)	1 (6.2)	2 (2.6)	3 (3.2)
*K. aerogenes*	0	5 (8)	5 (6.7)	0	7 (9.3)	7 (7.6)
*K. oxytoca*	0	0	0	0	2 (2.6)	2 (2.1)
*K. pneumoniae*	0	14 (22.5)	14 (18.9)	2 (12.5)	15 (20)	17 (18.6)
*Proteus* spp.	0	2 (3.2)	2 (2.7)	0	3 (4)	3 (3.2)
*P. aeruginosa*	1 (8.3)	26 (41.9)	27 (36.4)	3 (18.7)	28 (37.3)	31 (34)
*M. catarrhalis*	2 (16.6)	2 (3.2)	4 (5.4)	2 (12.5)	2 (2.6)	4 (4.3)
*H. influenzae*	7 (58.3)	8 (12.9)	15 (20.2)	9 (56.2)	12 (16)	21 (23)
*S. marcescens*	0	4 (6.4)	4 (5.4)	0	6 (8)	6 (6.5)
Total	13	70	83	19	90	109
Gram positive, number of targets detected (% ***)						
*S. agalactiae*	0	3 (4.8)	3 (4)	0	3 (4)	3 (3.2)
*S. pyogenes*	0	0	0	2 (12.5)	0	2 (2.1)
*S. aureus*	1 (8.3)	23 (37)	24 (32.4)	4 (25)	29 (38.6)	33 (36.2)
MSSA	1	11	12	3	15	18
MRSA	0	12	12	1	14	15
*S. pneumoniae*	6 (50)	4 (6.4)	10 (13.5)	6 (37.5)	7 (9.3)	13 (14.2)
Total	7	30	37	12	39	51
Total	20	100	120	31	129	160

* Positive results were ≥10^4^ for BAL, ≥10^5^ for ETA and NBL, 10^7^ for sputum. ** Positive results were ≥10^4^ for BAL, ETA and NBL and ≥10^6^ for sputum. *** Totals exceed 100% since many patients had more than 1 target found. Abbreviations: CAP—community acquired pneumonia, VAP—ventilator-associated pneumonia, FA—FilmArray, MSSA—Methicillin-sensitive *S*. *aureus*, MRSA—Methicillin-resistant *S. aureus*.

**Table 4:** Analytical performance of FA compared to culture using a strict and less strict sets of rules (Strict */Less strict **).

	FA(+)/Culture(+)	FA(+)/Culture(−)	FA(−)/Culture(+)	FA(−)/Culture(−)	Sensitivity (%)	Specificity (%)	PPV (%)	NPV (%)
Gram negative								
*E. cloacae*	7/7	1/5	2/2	118/114	77.7/77.7	99.1/95.8	87.5/58.3	98.3/98.3
*E. coli*	1/1	0/1	0/0	127/126	100/100	100/99.2	100/50.0	100/100
*A. baumannii*	0/0	1/2	1/1	126/125	0/0	99.2/98.4	0/0	99.2/99.2
*K. aerogenes*	2/4	2/2	2/0	122/122	50/100	98.4/98.4	50/66.6	98.4/100
*K. oxytoca*	0/1	0/1	1/0	127/126	0/100	100/99.2	-/50	99.2/100
*K. pneumoniae*	10/13	4/4	4/1	110/110	71.4/92.8	96.5/96.5	71.4/76.4	96.5/99.1
*Proteus* spp.	0/1	2/2	0/0	126/125	-/100	98.4/98.4	0/33.3	100/100
*P. aeruginosa*	22/24	0/2	2/0	104/102	91.6/100	100/98	100/92.3	98.1/100
*M. catarrhalis*	1/1	1/1	0/0	126/126	100/100	99.2/99.2	50/50	100/100
*H. influenzae*	2/2	11/17	1/1	114/108	66.6/66.6	91.2/86.4	15.3/10.5	99.1/99.1
*S. marcescens*	1/2	2/3	0/0	125/123	100/100	98.4/97.6	33.3/40	100/100
Total	46/56	24/40	13/5	1325/1307	77.9/91.8	98.2/97	65.7/58.3	99.0/99.6
Δ ***	+10	+16	−8	−18	+13.9	−1.2	−7.4	+0.6
								
Gram positive								
*S. agalactiae*	2/2	1/1	0/0	125/125	100/100	99.2/99.2	66.6/66.6	100/100
*S. pyogenes*	0/1	0/1	1/0	127/126	0/100	100/99.2	NA/50	99.2/100
*S. pneumoniae*	1/1	7/10	0/0	120/117	100/100	94.4/92.1	12.5/9.1	100/100
*S. aureus*	20/23	3/9	5/2	100/94	80/92	97.1/91.2	86.9/71.8	95.2/97.9
MSSA	9/11	2/6	4/2	113/109	69.2/84.6	98.3/94.8	81.8/64.7	96.6/98.2
MRSA	11/12	1/5	1/0	115/111	91.6/100	99.1/95.7	91.6/70.6	99.1/100
Total	23/27	11/21	6/2	472/462	79.3/93.1	97.7/95.6	67.6/56.2	98.7/99.5
Δ	+4	+10	−4	−10	+13.8	−2.1	−11.4	+0.8
								
Total	69/83	35/61	19/7	1797/1769	78.4/92.2	98.1/96.6	66.3/57.6	98.9/99.6
Δ	+14	+26	−12	−28	+13.8	−1.5	−8.7	+0.7
Resistance genes								
CTX-M	13/14	4/6	4/3	107/105	76.4/82.3	96.4/94.6	76.4/70	96.4/97.2
NDM	0/0	0/1	0/0	128/127	-	100/99.2	NA/0	100/100
IMP	0/0	0/0	0/0	128/128	-	100/100	-	100/100
KPC	0/0	0/0	0/0	128/128	-	100/100	-	100/100
OXA-48-like	0/0	0/0	0/0	128/128	-	100/100	-	100/100

* Strict set of rules: ≥10^4^ genome copies/mL for BAL, ≥10^5^ for ETA/NBL and ≥10^7^ for sputum. ** Less strict set of rules: ≥10^4^ genome copies/mL for BAL/ETA/NBL and ≥10^6^ for sputum. *** Difference between the strict and less strict sets of rules. Abbreviations: FA—FilmArray, PPV—positive predictive value, NPV—negative predictive value, NA—not applicable.

**Table 5:** Cases in which antimicrobials were avoided, started, or stopped as a result of FA testing.

Action Triggered by FA Result *n* (%)		Number of Patients	FA Results (*n*)		ABx Administered after FA Result	Corresponding Culture Results (*n*)	
Avoiding ABx 21 (28.0)		21 (100.0)	Negative FA		N/A	Normal flora (20) *H. influenzae* (1)	
Starting ABx 41 (54.7)	Patients not treated previously 34 (82.9)	18 (52.9)	*H. influenzae* (11) *S. pneumoniae* (6) MSSA (5) *M. catarrhalis* (2)	*K. pneumoniae* (2) *E. coli* (1) GBS (1) Negative FA (2)	ABx for community pathogens CRO (18)	MSSA (4) *K. pnuemoniae* (2) *E. cloacae* (2) *S. pneumoniae* (1) GBS (1) *E. coli* (1)	*A. baumannii* (1) *H. influenzae* (1) *M. catarrhalis* (1) *Citrobacter* spp. (1) *Aspergillus* spp. (1) Normal flora (8)
		7 (20.6)	*P.aeruginosa* (6) *E. coli* (1) *E. cloacae* (2) *H. influenzae* (1)	*K. pneumoniae* (2) MSSA (1) *A. baumannii* (1) *Serratia* spp. (1)	ABx for non-fermenters CAZ (5), TZP (2)	*P.aeruginosa* (3) *E. cloacae* (2) MSSA (1)	*K. pnuemoniae* (1) *Serratia* spp. (1) Normal flora (2)
		4 (11.8)	MSSA (3, one was sputum 10^4^) *H. influenzae* (2) *S. pneumoniae* (1) GAS (1) Negative FA (1)		ABx for MSSA CFZ (3), AMC (1)	MSSA (2) Normal flora (1)	
		1 (2.9)	*K. pneumoniae*, CTX-M (1)		ABx for ESBL ETP (1)	*K. pneumoniae* (CRO-Res)	
		4 (11.8)	MRSA (4, one was sputum 10^5^) *P. aeruginosa* (1) GAS (1)		ABx for MRSA VAN (4)	MRSA (2) *P. aeruginosa* (1) *Aspergillus* spp. (1) MSSA (1) Normal flora (1)	
	Patients in “ABx window” 7 (17.1)	1 (14.3)	MRSA, *P.aeruginosa*		VAN + TZP	MRSA, *P.aeruginosa*	
		1 (14.3)	MRSA, *E. cloacae* (ETA—10^4^), CTX-M		VAN	MRSA, *S. maltophilia*	
		1 (14.3)	MSSA, *K. pneumoniae*		CIP + CFZ	MSSA, *K. pneumoniae* (CRO-Sus)	
		1 (14.3)	*K. aerogenes*		TZP	*K. aerogenes* (CRO-Sus)	
		1 (14.3)	MRSA, *E. cloacae*, CTX-M		VAN + CIP	MRSA, *E. cloacae* (CRO-Res)	
		1 (14.3)	MRSA, *K. aerogenes*		VAN + MEM	*K. aerogenes* (CRO-Res) *C. striatum*	
		1 (14.3)	*P. aeruginosa*		CAZ	*P. aeruginosa*	
Stopping ABx 13 (17.3)		11 (84.6)	Negative FA (11)		TZP (2) CXM + AZM (1) CRO (4) CRO + LVX (2) LVX (1) CHL (1)	Normal flora/yeasts (10) N/A (1)	
		2 (15.4)	*K. aerogenes* (1) MRSA (1)		MEM (1) TZP (1)	*K. aerogenes* (1) MRSA (1)	

FA—FilmArray, ABx—Antibiotics, N/A—not available (no corresponding culture), ETA—endotracheal aspiration, CRO-Res—ceftriaxone resistance, CRO-Sus—ceftriaxone susceptible, MSSA—methicillin sensitive *S. aureus*, MRSA—methicillin resistant *S. aureus*, GBS—*Streptococcus agalactiae*, GAS—*Streptococcus pyogenes*, CRO—ceftriaxone, TZP—piperacillin-tazobactam, VAN—vancomycin, CFZ—cefazolin, ERT—ertapenem, CIP—ciprofloxacin, MEM—meropenem, CXM—cefuroxime, AZM—azithromycin, LVX—levofloxacin, CHL—chloramphenicol, CST—colistin, SXT—trimethoprim/sulfamethoxazole, VRC—voriconazole, LZD—linezolid, CAZ—ceftazidime, OXA—oxacillin, AMP—ampicillin, AMB—amphotericin B deoxycholate, AMK-amikacin.

**Table 6:** Cases in which antimicrobials were downgraded, upgraded, or left unchanged as result of FA testing.

Action Triggered by FA Result *n* (%)		Number of Patients	FA Results (*n*)	ABx Given before FA Results	ABx Given after FA Results	Culture Results (*n*)
Downgrading ABx 5 (6.8)		1	*E. cloacae, P. aeruginosa*	LVX	CIP	*P. aeruginosa*
		1	MSSA	TZP	OXA	*Citrobacter* spp.
		1	Negative FA	VAN + TZP	TZP	Normal flora
		1	MSSA	CRO	CFZ	MSSA
		1	Negative FA	CRO + LVX	CRO	Normal flora
Upgrading ABx 27 (37.0)	For *P. aeruginosa* coverage 13 (48.1)	13	*P. aeruginosa* (13) and: *K. pneumoniae* (3) *Serratia* spp. (3) MSSA (2) MRSA (1) *K. aerogenes* (2) *Proteus* spp. (2) *H. influenzae* (2) *E. cloacae* (1) *A. baumannii* (1) CRO-M (2)	CRO (5) VAN & LVX (1) VAN (1) CRO + AZM (1) LVX (1) CAZ (1) CHL (2) ERT + VAN (1) CXM (1)	TZP (8) MEM (1) MEM + VAN (1) MEM + CST (1) CRO + LVX (1) CIP (1)	*P. aeruginosa* (10), MSSA (2), MRSA (1), *K. pneumoniae* CRO-Res (1), *Providencia* spp. (1), *Roultella* spp. (1), *K. aerogenes* (1), Normal flora (1), N/A (1)
	For MRSA coverage 4 (14.8)	1	MRSA, *K. oxytoca*, CTX-M	CRO + AZM	SXT	MRSA, *K. oxytoca* CRO-Res
		1	MRSA, *K. oxytoca*, CTX-M	CRO	VAN + TZP	MRSA, *E. cloacae* CRO-Res
		1	MRSA	TZP	VAN + LVX	MRSA
		1	MRSA, *K. pneumoniae*	ERT	SXT	*K. pneumoniae* Non-CP-CRE
	For ESBL coverage 4 (14.8)	1	CTX-M, *E. cloacae*, *E. coli*, *Serratia* spp.	SXT	MEM	*Serratia* spp., *S. maltophilia*
		1	CTX-M, *E. cloacae*	AZM	TZP	*E. cloacae* CRO-Res
		1	CTX-M, *K. pneumoniae*	ERT	MEM + LZD	Yeast
		1	CTX-M, *K. pneumoniae*	TZP	MEM	*K. pneumoniae* CRO-Res
	Others 6 (22.2)	1	Negative FA	CST	AMB + VAN	N/A
		1	Negative FA	CFZ	SXT	Normal flora
		1	*E. cloacae* (Sputum 10^4^)	AZM	CIP	NA
		1	MSSA	TZP	TZP + CFZ	Normal flora
		1	*H. influenzae*	AMP	CRO	Normal flora
		1	*H. influenzae, S. pneumoniae*, MSSA	AMK (due to bacteremia)	CRO	MSSA
No change in ABx 41 (56.2)	Negative FA (*n* = 17)	17	Negative FA	CRO (9) TZP (2) CRO + VAN (1) CRO + LVX (1) LVX (1) VAN (1) VAN + VRC (1) CHL (1)	Same	Normal flora (9), MSSA (2), *S. maltophilia* (1), *Actinomyces* spp. (1), *C. striatum* (1), *Aspergillus flavus* (1), *P. aeruginosa* (1), *S. constellatus* (1)
	*P. aeruginosa* in FA (*n* = 8)	8	*P. aeruginosa* and: CTX-M (3) MRSA (1) MSSA (1) *Proteus* spp. (1) *K. pneumoniae* (1) *E. cloacae* (1) *K. aerogenes* (1)	TZP (2) CAZ (2) CAZ + CST (1) CST (1) VAN + TZP (1) MEM + VAN (1)	Same	*P. aeruginosa* (8) and *K. pneumoniae* CRO-Res (1), MRSA (1), *Citrobacter* spp. (1)
	CTX-M in FA (*n* = 3)	3	*E. cloacae* (1) *K. pneumoniae* (1) *Serratia* spp. (1)	ERT (2) LVX (1)	Same	*S. maltophilia* (1), Normal flora (1), *K. pneumoniae* CRO-Res (1)
	MRSA in FA (*n* = 2, one was sputum 10^4^)	2	MRSA and *S. pneumoniae* (2)	MEM + VAN (2)	Same	Normal flora (2)
	MSSA in FA (*n* = 2)	2	MSSA (1) MSSA and GBS and *S. pneumoniae* (1)	LVX (1) CRO (1)	Same	*S. maltophilia* (1), Normal flora (1)
	Others (*n* = 9)	9	*H. influenzae* (3) *K. pneumoniae* (3) *M. catarrhalis* (2) GBS (1) *S. pneumoniae* (1) *A. baumannii* (1) *E. cloacae* (1) *K. aerogenes* (1)	CRO (8) ERT (1)	Same	*K. pneumoniae* (2), *E. cloacae* CRO-Res & *K. pneumoniae* (1), GBS (1), *K. aerogenes* (1), *C. striatum* (1)

FA—FilmArray, ABx—Antibiotics, N/A—not available (no corresponding culture), ETA—endotracheal aspiration, CRO-Res—ceftriaxone resistance, CRO-Sus—ceftriaxone susceptible, Non-CP-CRE—non carbapenemase-producing carbapenem resistant *Enterobacteriaceae*, MSSA—methicillin sensitive *S. aureus*, MRSA—methicillin resistant *S. aureus*, GBS—*Streptococcus agalactiae*, GAS—*Streptococcus pyogenes*, CRO—ceftriaxone, TZP—piperacillin-tazobactam, VAN—vancomycin, CFZ—cefazolin, ERT—ertapenem, CIP—ciprofloxacin, MEM—meropenem, CXM—cefuroxime, AZM—azithromycin, LVX—levofloxacin, CHL—chloramphenicol, CST—colistin, SXT—trimethoprim/sulfamethoxazole, VRC—voriconazole, LZD—linezolid, CAZ—ceftazidime, OXA—oxacillin, AMP—ampicillin, AMB—amphotericin B deoxycholate, AMK-amikacin.

## Data Availability

Data may be provided per request from the authors.
